# The high resistance loop (H-loop) technique used for all-inside arthroscopic knotless suprapectoral biceps tenodesis: A case series

**DOI:** 10.3389/fsurg.2022.917853

**Published:** 2022-09-14

**Authors:** Min Zhou, Chuanhai Zhou, Dedong Cui, Yi Long, Jiang Guo, Zhenze Zheng, Ke Meng, Jinming Zhang, Jingyi Hou, Rui Yang

**Affiliations:** Department of Orthopaedic Surgery, Sun Yat-sen Memorial Hospital, Sun Yat-sen University, Guangzhou, China

**Keywords:** biceps tenodesis, H-loop, all-inside, arthroscopy, functional outcomes

## Abstract

**Introduction:**

Suprapectoral tenodesis is a common technique for the treatment of long head biceps tendon lesions. However, so far, there is no gold standard treatment in all-inside arthroscopy. The purpose of the present study was to introduce and evaluate the functional outcomes of an innovative, all-inside arthroscopic high resistance loop (H-loop, high resistance to tissue cutout and 360° grasping of the tendon) technique for long head of biceps (LHB) tenodesis.

**Method:**

From September 2020 to March 2022, a series of cases of 32 consecutive patients (28 rotator cuff tear with LHBT pathology and 4 superior labrum anterior-posterior (SLAP) tears which including 2 type II and 2 type IV) who received LHB tenodesis using all-inside arthroscopic high resistance loop technique were included in this study. The American Shoulder and Elbow Surgeon Score (ASES), Visual Analog Scale (VAS), Simple Shoulder Test Score (SST), Constant–Murley scores, and University of California at Los Angeles Scoring System (UCLA) were used to evaluate the clinical outcomes of patients in preoperative and final follow-up. Meanwhile, postoperative complications were also observed.

**Result:**

32 patients (14 women and 18 men, average age was 55.7 years) underwent all-inside arthroscopic knotless suprapectoral biceps tenodesis using the H-Loop stitch technique. The mean time of follow-up was 16.2 ± 2.6 months. The ASES, VAS, Constant–Murley, SST, and UCLA scores improved from 51.5 ± 15.8, 5.5 ± 1.6, 57.8 ± 14.7, 5.0 ± 2.8, and 16.1 ± 3.8 preoperatively, to 89.1 ± 7.5, 1.0 ± 0.8, 87.3 ± 5.5, 10.4 ± 1.5, and 31.3 ± 2.6 in the last follow-up, respectively (*p* < 0.001). During the follow-up, no patients in this study experienced postoperative complications such as infection of the wound, injury of nerves, and hardware failure; no patients required revision after their operation. In addition, none of the patients had cramping or a “Popeye” deformity during follow-up.

**Conclusion:**

This article presents an innovative, all-arthroscopic H-loop technique for LHB tenodesis. This technique for LHB tenodesis showed favorable functional and cosmetic outcomes, as well as high satisfaction rates. Due to its simplicity of operation and satisfactory preliminary clinical outcomes, H-loop technique is perhaps another option to choose in all-inside arthroscopic LHB tenodesis.

## Introduction

Lesions of the long head of the biceps tendon (LHBT) usually lead to pain of the shoulder and disability, and more than 60% of patients have a rotator cuff tear (1). Tenotomy and tenodesis are effective but controversial techniques for the treatment of LHBT lesions; however, there was no difference in functional results between tenotomy and tenodesis in recent studies (2).

LHBT tenotomy is technically easier to perform and is associated with early amelioration of post-operative pain, but the incidence of cosmetic deformities is higher (25%–62%) (3–5). However, in young or elderly patients with higher requirements, tenodesis is suggested to reduce the occurrence of cramping, “Popeye” deformity, and weakness in the biceps brachii (6, 7).

To overcome this problem, several LHBT fixation techniques have been described, including open and arthroscopic techniques, which provided satisfactory results without clinically significant differences (8, 9). A study performed by Gombera et al. compared arthroscopic with open LHB tenodesis (10). The results showed similar clinical outcomes between the arthroscopic suprapectoral tenodesis and open subrapectoral tenodesis groups. However, they announced that open subpectoral tenodesis may be associated with an increased risk of complications because of its requirement to expose more tissue. For open subrapectoral tenodesis, some complications have been reported, such as neurovascular injury (11, 12), fractures (13–16), and deep infection (17, 18); but the complications above can be mitigated by adopting an arthroscopic approach (19–21).

Numerous types of tenodesis techniques can be used to treat LHBT pathology, such as suture anchor, interference screw, and cortical button (22–24). Biomechanics studies have evaluated these different tenodesis techniques and showed slight differences; but there was no difference in clinical outcomes (6, 25, 26). Therefore, using a relatively simple and safe fixation method is the best choice for tenodesis.

Regarding tenodesis with suture anchor *via* arthroscopy, the Lasso-Loop stitch was commonly used (27–31).This technique provided strong tissue grasping ability, which was equivalent to interference screws (32). However, compared to the Krakow Stitch, Lasso-Loop stitch had a critical defect where the uneven load distribution of sutured tendon might lead to poor tendon fixation strength (33).

To solve these specific problems, a new Loop stich, high resistance loop (H-Loop) (invented by Jingyi Hou), was developed to provide 360° grasping of the tendon and to correct unevenness of sutured tendon load distribution. In this study, the circumferential high resistance loop grasping LHBT was introduced. The purposes of this research were: (i) to present the H-Loop stitch technique in arthroscopic LHB tenodesis; (ii) to assess the preliminary outcomes in patients with LHB tenodesis using the H-Loop stitch technique.

## Materials and methods

### Patient selection

The study was approved by the Ethics Committee of Sun Yat-sen Memorial Hospital of Sun Yat-sen University (SYSEC-KY-KS-2021-303), and abided by the Declaration of Helsinki and the Guidelines for Good Clinical Practice. A retrospective case series data on patients between September 2020 and March 2022 who have undergone all-inside arthroscopic knotless suprapectoral biceps tenodesis with H-Loop stitch by the senior author (R.Y.) was prospectively and consecutively collected.

The inclusive criteria included: (i) patients over 18 years of age who were diagnosed with LHBT pathologies; (ii) all arthroscopic biceps tenodesis with H-Loop stitch technique performed together with arthroscopic repair of a rotator cuff tear or superior labrum anterior-posterior (SLAP) tears; (iii) magnetic resonance imaging demonstrating LHBT pathologies that did not improve with conservative treatment after 6 months; (iv) shoulder functions were recorded and the pre- and post-operative last follow-up were compared using the American Shoulder and Elbow Surgeon Score (ASES) (34), Visual Analog Scale (VAS), Simple Shoulder Test Score (SST), Constant–Murley scores (35), and University of California at Los Angeles Scoring System(UCLA) (36). Exclusion criteria included: revision surgery after rotator cuff repair, shoulder osteoarthritis, adhesive capsulitis, and individuals with marked deformity and/or neuromuscular diseases.

According to the inclusion and exclusion criteria, a total of 32 consecutive patients (14 women, 18 men) who were diagnosed with LHBT lesions (28 rotator cuff tear with LHBT pathology and 4 superior labrum anterior-posterior (SLAP) tears which including 2 type II and 2 type IV) and were surgically treated with the all-inside arthroscopic knotless suprapectoral biceps tenodesis with H-Loop stitch were enrolled in this study. Before the operation, all patients received extensive shoulder examination, including physical, x-ray, and MRI examination, and were diagnosed with LHBT lesion with or without other concomitant diseases of the shoulder.

### Surgical technique

#### Anesthesia and patient positioning

After general anesthesia, the patient was placed in a lateral decubitus position on the operation table. Then, the operative shoulder was placed at 20° forward flexion and 35°–45° abduction. Continuous traction was applied through the ipsilateral upper extremity to gain a larger operative space in the glenohumeral joint.

#### Diagnostic arthroscopy

**Figure 1:**
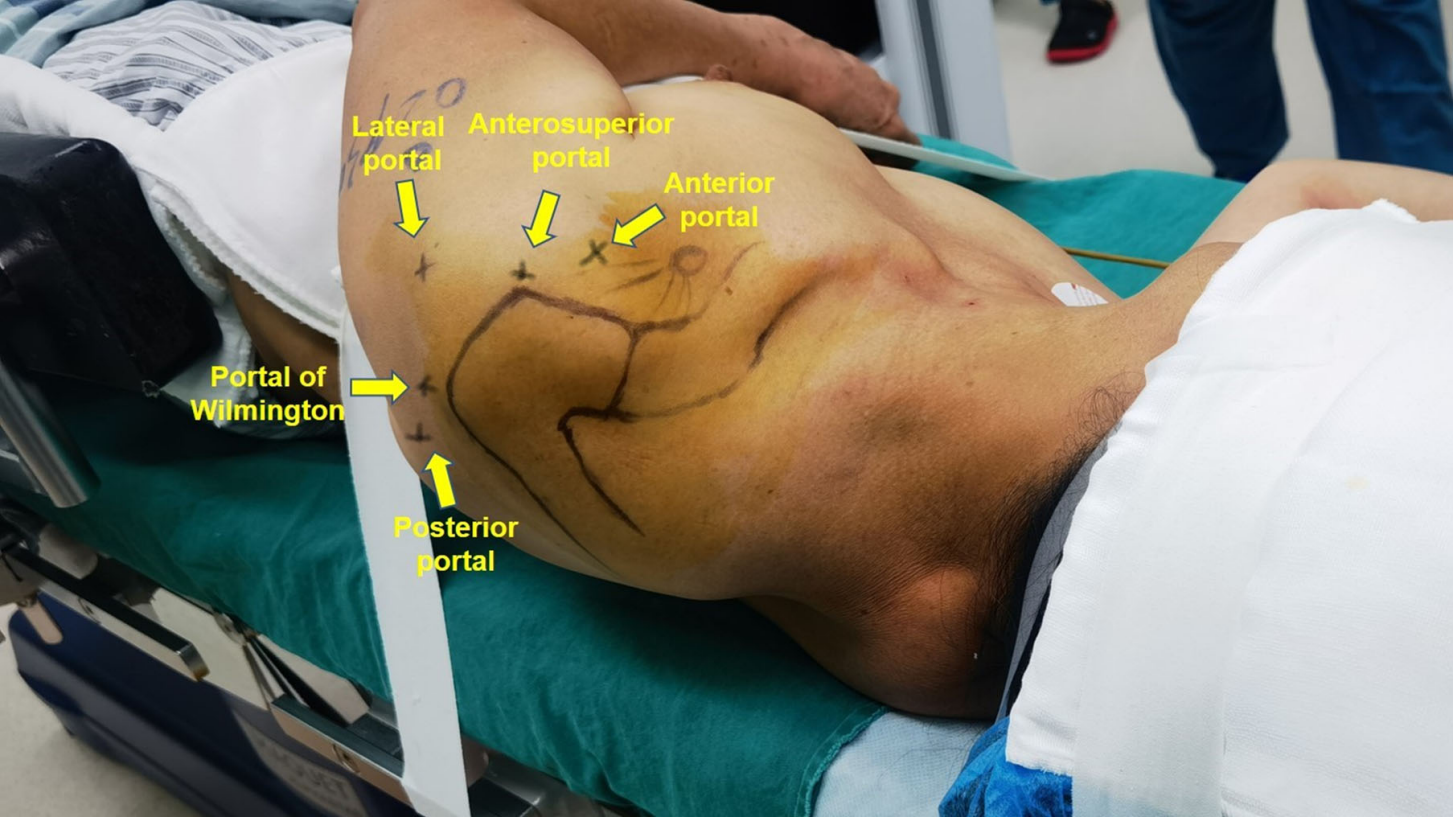
The standard anterior portal, lateral portal, posterior portal, anterosuperior portal and, portal of Wilmington had been marked.

The standard routine portal was established in turn: posterior, anterior, and anterolateral portal. The glenohumeral joint and long head of the biceps tendon was examined thoroughly through the posterior portal. To clarify the pathological changes of the intertubercular groove part of the LHBT, the LHB needs to be pulled toward the intra-articular area using probes from the anterior approach. Hence, an anterior approach was established under arthroscopic surveillance. Additionally, a lateral portal was established by spinal needle for rotator cuff repair (Figure 1).

#### The H-loop stitch technique for long head of the biceps tendon

**Figure 2:**
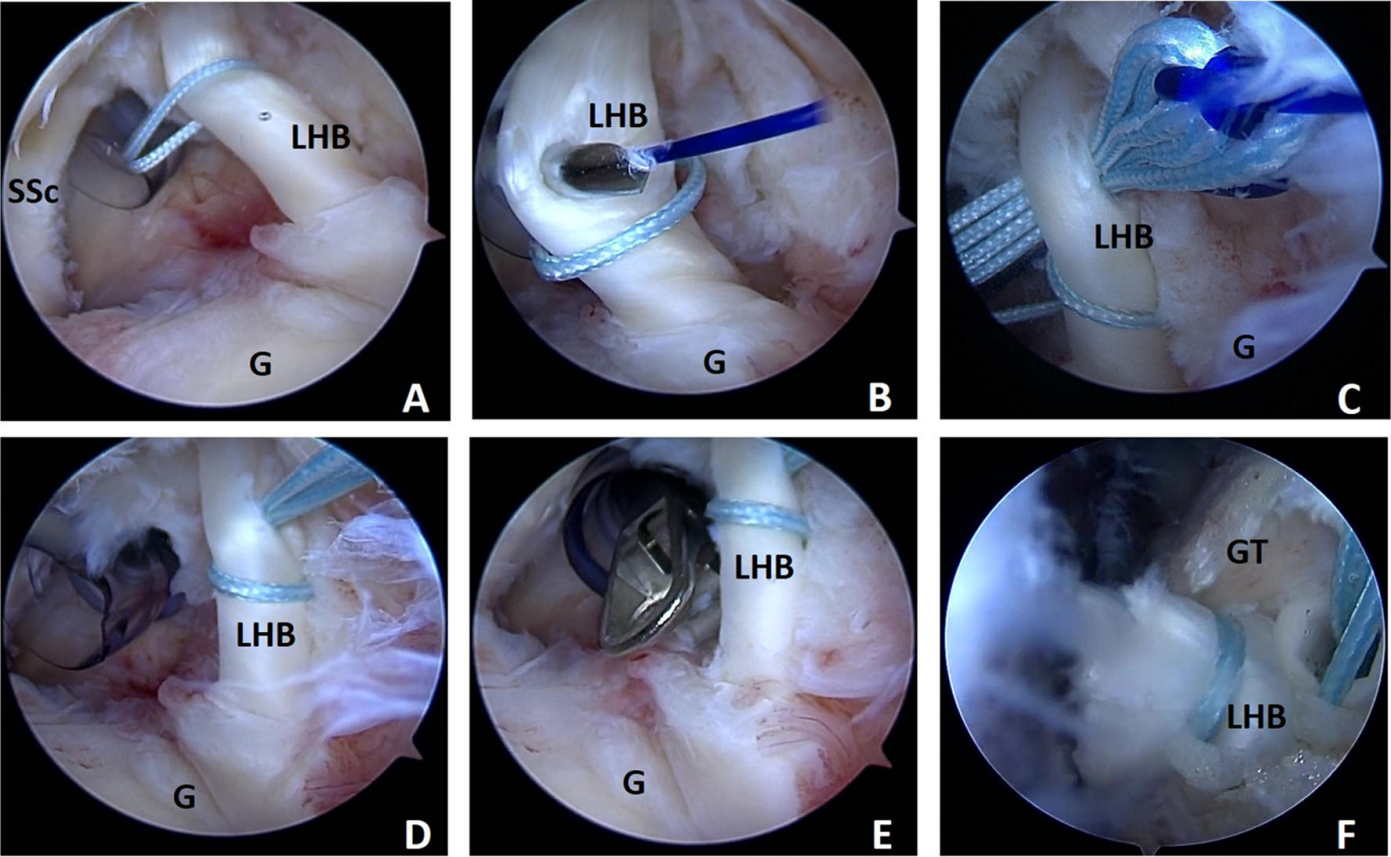
Demonstration of the high resistance loop (H-Loop) stich technique arthroscopic surgical in shoulder as viewed from the posterior portal with the patient in the lateral decubitus position using a 30° scope. (**A**) a 2# Fiber Wire suture (Arthrex, Naples, FL) was folded in half and inserted into glenohumeral joint and construct a loop hitching around the LHBT; (**B**) A Suture Lasso is pierced through the midportion of the LHBT just distal of the loop to advance a 0# PDS II Suture, as a guiding suture. (**C**) The two ends of Fiberwire suture were brought into midportion of the LHBT just distal of the loop with PDS suture; (**D**) A novel, self-locking, high-resistant rip-stop loop configuration was constructed; (**E**) The origin of LHBT was detached with a curved arthroscopic scissor. (**F**) The two free ends of the suture were pressed into the guide hole with a push lock anchor suture (Arthrex, Naples, FL) at the intertubercular groove.

Once the LHBT tenodesis was determined, a FiberWire suture (2#, Arthrex, Naples, FL) was folded in half and inserted into the capsule with the suture grasper from the anterior portal. The suture was first released at the superior aspect of the tendon near the insertion point. One free end of the suture was held on to outside the arthroscope and the suture was grasped from the inferior aspect of the tendon in the arthroscope, while another end of the suture was pulled outside the arthroscope to construct a loop hitching around the LHBT. A SutureLasso SD 90° (Arthrex, Naples, FL), the suture shuttle device, was pierced through the midportion of the LHBT just distal to the loop through the anterior portal to advance a 0# PDS II (polydioxanone suture) (Ethicon Inc; Johnson / Johnson, Somerville, NJ) as a guiding suture. Subsequently, the SutureLasso was retrieved, and the end of the PDS suture in the capsule was grasped out through the posterior portal. A tight overhand knot was tied on the two ends of the FiberWire suture with the PDS suture. Finally, the PDS suture was pulled out from the posterior portal, helping the two suture ends shuttle the tendon. Then, a novel, self-locking, high-resistant loop configuration was constructed after removing the PDS suture (Figures 2A–E).

#### Long head biceps tenodesis

A punch forcep was inserted into the articular cavity through the anterior portal to detach the LHBT at the insertion site on the superior labral junction. The arthroscope was subsequently shifted to the subacromial space. The transverse ligament was completely cut and the LHBT was thoroughly exposed in the bicipital groove with radiofrequency ablation through the anterolateral portal. The tendon was provoked with the probe to further expose the bicipital groove and thorough debridement was performed with an arthroscopic burr to refresh the intended anchor site. A pilot hole was drilled *via* the bicipital groove approach to the capsule border perpendicularly with a punch for the 4.75 mm SwiveLock C Anchor (Arthrex, Naples, FL). Two free ends of the suture were loaded into the eyelet of the anchor. Finally, the anchor was placed into the pilot hole, and the tail sutures under the anchor were cut off (Figure 2F).

### Rehabilitation

The arm was placed in 30° abduction with an abduction brace for 6 weeks after operation. At this stage, patients were allowed to perform gentle pendulum exercises and elbow/wrist range of motion exercises under the conduct of a physical therapist. After 6 weeks, active exercises of the shoulder and biceps were initiated. 12 weeks after the operation, biceps strengthening programs were started.

### Clinical outcomes measure

The clinical results of preoperative and postoperative final follow-up were evaluated by the following scales: ASES, VAS, Constant–Murley, SST, and UCLA score. And the cramps, “Popeye” deformity, tenderness of the bicipital groove, pain during the performance of the Speed test were also evaluated. In the final follow-up, the satisfaction of this technique had been evaluated.

### Statistical analysis

SPSS 20.0 (IBM, Armonk, USA) was used for statistical analysis. The ASES, VAS, Constant–Murley, SST, and UCLA scores between preoperative and final follow-up were compared with Wilcoxon signed-rank test for non-parametric data sets, where a *P* value of <0.05 was considered statistically significant. The categorical variables were measured by proportion.

## Results

### General outcomes

The age of patients ranged from 34 to 68 years, with a mean of 55.7 years. All cases have been followed up recently. The mean follow-up was 17 months (range, 12–20 months). In this cohort of patients, the pathology of LHBT was defined during arthroscopic surgery, and the indications for biceps tenodesis included partial tear of the LHB, symptomatic LHB tendinitis, chronic LHB tendinopathy, type 2 and type 4 SLAP tears, and subluxated or dislocated LHB with associated rotator cuff tear. Of the 32 patients, 30 (93.8%) had at least 1 additional intervention during biceps tenodesis.

### Functional outcomes

#### ASES score

The clinical scores of the ASES showed that the mean preoperative score was 51.5 (SD = 15.8) and the mean score during the final postoperative follow-up was 89.06 (SD = 7.48), with a statistically significant difference (*p* < 0.001).

#### VAS score

The clinical scores of the VAS scale showed that the mean preoperative pain score was 5.5 (SD = 1.6) and the mean pain score during the final postoperative follow-up was 0.97 (SD = 0.82), with a statistically significant difference (*p* < 0.001).

#### Constant–murley score

The clinical scores of Constant–Murley showed that the mean preoperative score was 57.8 (SD = 14.7) and the mean pain score during the final postoperative follow-up was 87.3 (SD = 5.5), with a statistically significant difference (*p* < 0.001).

#### SST score

The clinical scores of the SST showed that the mean preoperative score was 5.0 (SD = 2.8) and the mean pain score during the final postoperative follow-up was 10.4 (SD = 1.5), with a statistically significant difference (*p* < 0.001).

#### UCLA score

The clinical scores of the UCLA showed that the mean preoperative score was 16.06 (SD = 3.8) and the mean pain score during the final postoperative follow-up was 31.3 (SD = 2.6), with a statistically significant difference (*p* < 0.001).

The procedure has high patient satisfaction; 62.5% (12/32) of patients classified the clinical outcome as excellent and 37.5% (12/32,) as good (residual gentle tenderness in intertubercular/bicipital groove). At the final follow-up after surgery, all patients returned to their daily lives without limitations.

### Complications

During the follow-up, no patients in this study experienced postoperative complications such as infection of the wound, injury of nerves, and hardware failure; no patients required revision after their operation. In addition, none of the patients had cramping or a “Popeye” deformity during the final follow-up after surgery.

## Discussion

The present study was designed to introduce the all-inside arthroscopic knotless suprapectoral biceps tenodesis with H-Loop stitch and to access the preliminary outcomes in patients with LHB tenodesis using the H-Loop stitch technique. The principle finding of the present study presented good clinical results and acquired a high rate of patient satisfaction. The functional scores and pain of patients demonstrated significant improvement. No patients in this study experienced nerve damage and re-operation postoperatively. All-inside arthroscopic knotless suprapectoral biceps tenodesis with H-Loop stitch is a treatment option for LHBT disorders.

Optimal tenodesis remains a contentious issue, with current tenodesis hardware options including a wide range of implants such as interference screws, cortical buttons, and suture anchors (22–24). Due to the high biomechanical stability and good clinical outcomes, the interference screw technique has been widely used in LHB tenodesis (18, 37). However, some serious adverse events related to interference screw LHB tenodesis have also been reported successively, including humeral shaft fractures, tendon injuries within bone tunnels, and local reactions for bioabsorbable screws (38, 39). To further investigate the complications of the interference screw bone tunnel tenodesis, relevant biomechanical studies have been recently performed. The results of this study showed similar ultimate failure loads and stiffness of all-suture anchor and interference screw constructs. However, in the torsion test, implant-related fractures occurred in only 29% of the suture anchor tenodesis structures versus 170% of the interference screw tenodesis structures (25). In clinical practice, the interference screw bone tunnel tenodesis technique has been confirmed to have a higher incidence rate of postoperative cosmetic deformities and revision surgery as compared with the all-suture anchor bone surface tenodesis technique (26). Therefore, the suture anchor bone surface tenodesis technique was proposed as a reasonable option to reduce these risks. The current preliminary clinical results may support these findings, because neither humeral shaft fractures nor cosmetic deformities were observed at the final follow-up.

The suture anchor fixation technique for tenodesis is a simple and relatively safe fixation method and is widely accepted by surgeons. Lafosse first described the “Lasso-loop” technique to tenodesis in all-arthroscopic surgery (27). Additionally, other investigators have found “Lasso-loop” LHB tenodesis with suture anchor achieved more stronger and securer than the LHB tenodesis with interference screws (32). However, other biomechanical testing has demonstrated that the “Lasso-loop” technique holds only a portion of the tendon, which results in an unbalanced suture tendon load distribution, and does not avoid failure of tendon fixation due to the cutting effect of sutures on the tendon (33). To address these specific problems, Sebastian et al. introduced the Lasso Loop 360 technique to provide secure fixation and improved biomechanical properties. Compared with the Lasso-loop technology, the maximum failure load, displacement, and stiffness of Lasso Loop 360 have been improved (40). However, this technique did not solve the problem of tendon cutting by suture; meanwhile, the results of this technique were only ideal for experimental conditions and has not been clinically used and confirmed.

**Figure 3:**
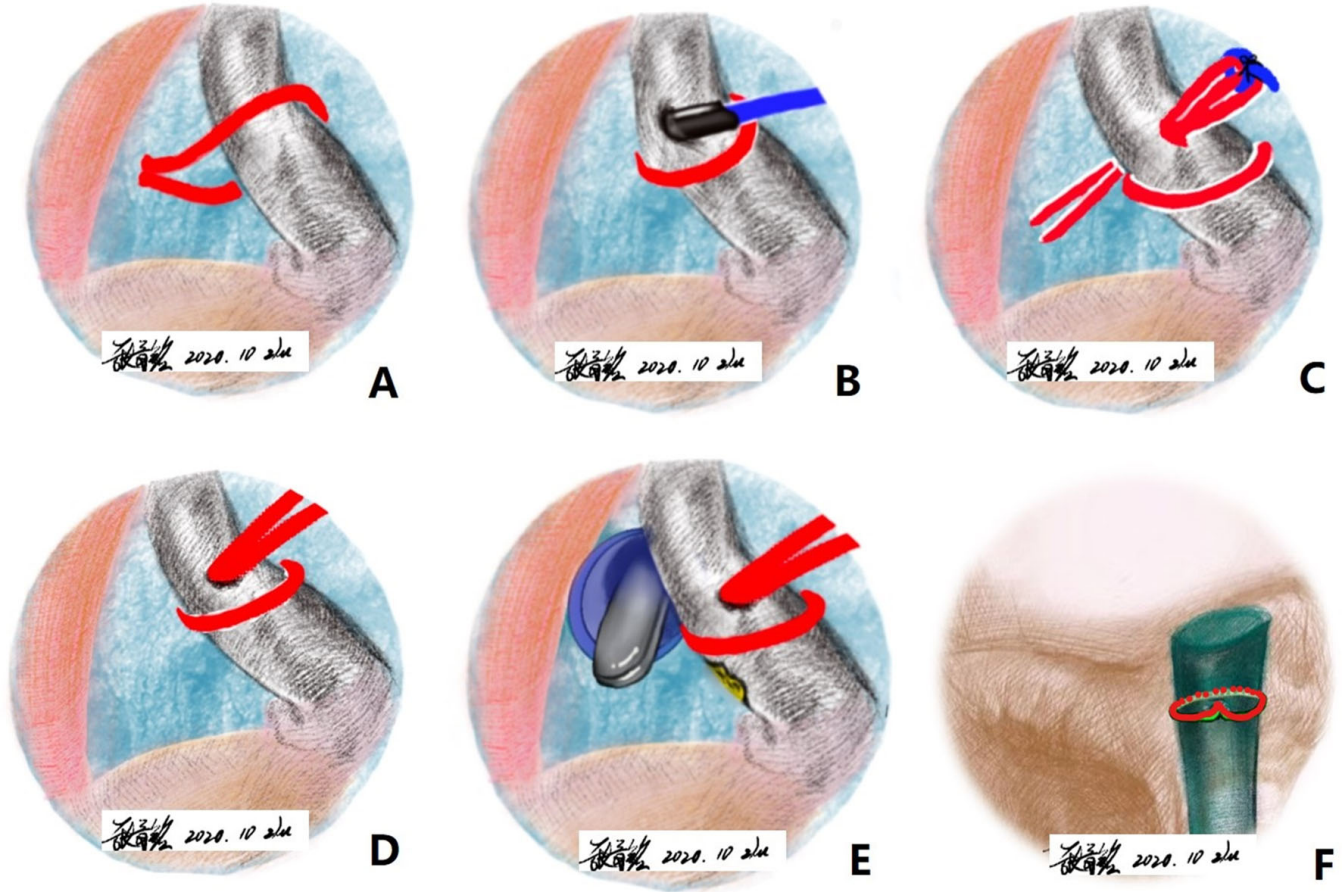
Illustrations summarizing the steps required to create H-LOOP tenodesis of LHB.

All-inside arthroscopic knotless suprapectoral biceps on-lay tenodesis with H-Loop stitch, which is a novel, total-grasp, self-locking, and high resistance stitch technique has obtained satisfactory clinical results in this case series. The H-loop stitch technique emphasizes the grasping of the whole biceps longus tendon, and not only demonstrates strong grip organization but also acts as a self-tightening force under stressful conditions (41). In addition, this suture loop opposes each other with the free limb of the suture, which can function as a rip-stop to prevent cutting of the tendon at the suture interface (42). The advantages of this technique are simple operation, excellent visualization, and easy to be taught and repeated under arthroscopy. At the same time, it is a cost-effective technology, because: (1) It is a simple and time-consuming technology, which saves operation time; (2) The two suture limbs can be pressed under the lateral row handling the rotator cuff without additional implants, which also reduces cost by eliminating the need for additional implants for tenodesis; (3) There is no need for an extra instrument, which can be completed with a simple lasso. In addition, this technique could be completed under arthroscopy, does not require additional separate incisions and may reduce surgical morbidity. A schematic of the steps of H-loop stitch is summarized in Figure 3. Technology-related tips and tricks are detailed in Table 1. Complete advantages and disadvantages are listed in Table 2.

**Table 1:** Technical pearls and pitfalls of H-loop tenodesis of LHB.

• A suitable anterior portal that facilitates access above and below the biceps tendon.
• The anterior portal placement cannot be placed close to the proximal aspect of biceps sulcus.
• Avoid repeatedly using the Lasso to pass through the biceps tendon to prevent injury.
•The suture of H-loops as close to insertion on labrum as possible (to allow hypotonic tenodesis).
•The intertubercular sulcus should thoroughly be debrided during the operation to reduce the occurrence of residual pain.
•The suture of H-loops should be more than 1 cm away from the incisal margin to reduce potential for suture loop pull out.

**Table 2:** Advantages and disadvantages of H-loop tenodesis of LHB.

Advantages	Disadvantages
• This technique has the advantages of simple process flow, all-arthroscopic operation under direct visualization from posterior portal.	• New learning curve for surgeons.
• This technique has an excellent visualization, and easy to be taught and repeated by surgeons.	• If the H-loop suture is placed too close to the incisal margin of the biceps, there is a possibility risk for suture pullout off the proximal biceps intraoperatively or postoperatively.
• This is a knotless and time-efficient technique, which save the operating room time;	• Insufficiency of clinical comparison data.
• This technique does not required extra incision, which may decrease tissue injury and perioperative infection.	

The following limitations of our study must be considered. First, this tenodesis technique has potential limitations similar to those of other all-arthroscopic tenodesis techniques. This is because this technique is simply performed to fix the LHBT at the proximal part of the bicipital groove. Because thorough debridement of the intertubercular groove is not performed during the procedure, it is possible that the remnant of lesional tissue leads to residual anterior shoulder pain^25^. Secondly, the small sample size and short follow-up duration might not be enough to obtain accurate outcomes. Hence, a large sample size and longer follow-up durations are needed to obtain more precise conclusions. Thirdly, the study is short of an appropriate control group, where a randomized design comparing the H-loop tenodesis technique with the conventional tenodesis technique is required. Fourthly, patients underwent rotator cuff repair and LHB tenodesis, which confounded our results. Fifthly, data of biomechanics are lacking, and the mechanical superiority of this technology needs to be further confirmed by biomechanics in the future.

## Conclusion

This article presents an innovative, all-arthroscopic H-loop technique for LHB tenodesis. Although the biomechanics of this tendon fixation technique have not been studied, it has shown favorable functional and cosmetic outcomes. Due to its simplicity of operation and satisfactory preliminary clinical outcomes, all-inside arthroscopic knotless suprapectoral biceps tenodesis with H-Loop stitch is another treatment option for LHBT disorders. However, the mechanical superiority of this technology needs to be further confirmed by biomechanics, and long-term clinical outcomes should have to follow to confirm the sustainable success of this promising technique.

## Data Availability

The raw data supporting the conclusions of this article will be made available by the authors, without undue reservation.
